# Smart Wearables in Healthcare: Signal Processing, Device Development, and Clinical Applications

**DOI:** 10.1155/2018/1696924

**Published:** 2018-10-09

**Authors:** Chengyu Liu, Feng Liu, Li Zhang, Yi Su, Alan Murray

**Affiliations:** ^1^State Key Laboratory of Bioelectronics, Jiangsu Key Laboratory of Remote Measurement and Control, School of Instrument Science and Engineering, Southeast University, Nanjing 210096, China; ^2^University of Queensland, Brisbane, Australia; ^3^University of Northumbria, Newcastle upon Tyne, UK; ^4^Institute of High Performance Computing, A∗STAR, Singapore; ^5^Newcastle University, Newcastle upon Tyne, UK

Recently, smart wearables, typically as wearable electrocardiogram (ECG), electroencephalography (EEG), electromyography (EMG), blood pressure (BP), photoplethysmography (PPG), heart sound, respiration, sleep, and motion monitoring, have been gaining a significant role in the field of healthcare and are looking to be a big and promising market in the technology industry. They are scientifically and clinically useful for better monitoring of real-time, long-term, and dynamic physiological and pathological processes, thus providing opportunities for the development of new diagnostic and therapeutic techniques. These could be expedient for the management of chronic illnesses, such as cardiovascular disease (CVD), sleep disorders, emotional problems, cognitive impairment, and functional decline, as well as for healthcare applications for special populations, such as for the aged, pregnant women, athletes, and astronauts. The mainstream in smart wearables research is moving towards more sophisticated methodologies based on clinical “big data,” artificial intelligence (AI), advanced signal processing, service robots, and networks, as well as more robust signal acquisition approaches.

In this context, many researchers have addressed recent technology advances in signal processing and device development for smart wearables, as well as the implementation of these technologies for clinical applications. The processing and analysis of wearable physiological signals (ECG, EEG, EMG, BP, PPG, etc.) is a key issue for smart wearable devices. The preliminary work mainly includes dynamic signal quality assessment, signal transformation and decomposition, feature extraction and selection, and the following machine learning-based methods.

Y. Ning and colleagues decomposed multichannel surface electromyography (sEMG) signals into their constituent motor unit action potential (MUAP) trains. A combination method of measurement correlation (MC) and linear minimum mean square error (LMMSE) was proposed, named as MC-LMMSE, which was validated on both simulated and experimental electrode array sEMG signals. This study showed that the MC-LMMSE method can extract a relatively large number of MUs with strong robustness to noise.

As PPG technology has been widely applied to wearable sensors, L. Wang et al. developed a method for automatically estimating systolic blood pressure (SBP) and diastolic blood pressure (DBP) based only on a PPG signal. In this study, a multitaper method (MTM) was used for feature extraction and an artificial neural network (ANN) method was used for SBP and DBP estimation, obtaining a relatively high accuracy of BP estimation with an absolute error of 4.02 ± 2.79 mmHg for SBP and 2.27 ± 1.82 mmHg for DBP.

QRS complex location is important and even essential for ECG signal processing. F. Liu et al. performed a systematic evaluation work on ten widely used and high-efficient QRS detection algorithms, aiming at verifying their performances and usefulness in different application situations, especially in the dynamic noisy ECG environment. Four experiments were carried out on six internationally recognized databases. For the clean clinical ECG signals, the majority of the QRS detectors reported high-level detection accuracies, whereas the accuracy of all algorithms significantly decreased for poor signal quality ECG signals. Thus, some special preprocessing and postprocessing procedures are needed. In addition, the QRS detector needs to be carefully selected in special situations, such as paced rhythm ECGs. This study offers a reference for selecting from the existing algorithms.

Clinical applications for chronic illnesses detection, including cardiac arrhythmia, hypertension, heart failure, sleep disorder, emotional problem, cognitive impairment, and functional decline, are the main target. The automatic detection and diagnosis algorithms for special diseases are the soul of clinical application. Typical works included in this special issue are summarized as follows:

X. Xu et al. proposed a new framework for automatic atrial fibrillation (AF) beat identification, which combines modified frequency slice wavelet transform (MFSWT) and convolutional neural networks (CNNs). This work achieved a relatively high accuracy of 84.9%. The study indicated that it was possible to accurately identify AF or non-AF ECGs from a short-term signal episode.

Obstructive sleep apnea (OSA) is a major breathing-related sleep disorder. Y. Fang et al. proposed a novel sleep respiratory rate detection based on the characteristic moment waveform (CMW) method. This method could detect sleep respiratory rate accurately. In addition, the apnea sections can be detected by the sleep respiratory rate curve with a given threshold, and the time duration of the segmentation of the breath can be calculated for detailed evaluation of the OSA state.

D. V. Phan and team evaluated the relationship between daily physical activity (DPA) and memory capacity, as well as the association between daily activity and attention capacity, using spatial span test (SST) and trail making test (TMT). The study showed that the short-term effects of very active time duration (VATD) and calories burnt on the day are significantly and negatively associated with memory and attention capacities of college students.

Intelligent health monitoring systems combining wearable technologies for health monitoring and disease diagnosis also aroused widespread concern among researchers.

K. Guan et al. designed a remote health monitoring system for the elderly. This proposed system consisted of three parts: smart clothing, a smart home gateway, and a health care server. The system could monitor the ECG signals and motion signals of the elderly, and has the potential to provide long-term and continuous home healthcare monitoring services.

For gastrectomy patients requiring dietary support, K. Taniguchi et al. developed a chewing-count measurement device, named wearable reliable chewing-count (RCC), using an earphone-type sensor to display the information on a tablet terminal in real time. This earphone-type RCC measurement device could experimentally distinguish chewing from other actions. It can catch chewing actions and accurately count the number of chews with high probability.

Based on a piecewise three-segment sit-to-stand (STS) biomechanical model and a double-sensor difference algorithm, K. Liu and his group proposed an original approach for noninvasive estimation of lower limb joint moments for analysis of STS rehabilitation training with only inertial measurement units. This work presented joint kinematic and kinetic analysis using a customized wearable sensor system composed of accelerometers and gyroscopes. Compared with a referenced camera system, the proposed system was evaluated on five healthy subjects and five patients in rehabilitation. The results showed that the newly developed system was available for spatiotemporal analysis of STS task with fewer sensors and a high degree of accuracy, and can be used as a reference for rehabilitation training or feedback for the control of a powered exoskeleton system.

Low power- and energy-efficient hardware is still the obstacle for long-term real-time wearable devices. K. Luo et al. developed a digital compressed sensing- (CS-) based single-spot Bluetooth ECG node to deal with this challenge. Each node consisted of an ultra-low-power analog front-end, a microcontroller, and a Bluetooth 4.0 communication module. A periodic sleep/wake-up scheme and a CS-based compression algorithm were implemented in each node. This scheme can reduce the airtime over energy-hungry wireless links. The energy consumption of the proposed node is 6.53 mJ, and the energy consumption of the radio has a decrease of 77.4%.

Overall, the development of smart medical wearables still has far to go, from hardware devices (electrode and sensor design) to data processing and analysis. There is much meticulous work still to be done, including wearing comfort, energy consumption, database annotation, signal quality, building standards, and more. Even for ECG it would be an interesting study to see specifically how a multielectrode system responded to very specific situations, such as movement (always a current problem, although different electrode positions are likely to respond differently), body position, and heart rate. It is important to get beyond overall accuracy. Anyway, the gradual improvement of smart medical wearable devices will contribute a tremendous amount of power to human healthcare.

